# Hallmarks of Aging: An Autophagic Perspective

**DOI:** 10.3389/fendo.2018.00790

**Published:** 2019-01-09

**Authors:** María Carolina Barbosa, Rubén Adrián Grosso, Claudio Marcelo Fader

**Affiliations:** ^1^Laboratorio de Biología Celular y Molecular, Instituto de Histología y Embriología (IHEM), Universidad Nacional de Cuyo, CONICET, Mendoza, Argentina; ^2^Facultad de Odontología, Universidad Nacional de Cuyo, Mendoza, Argentina

**Keywords:** autophagy, ROS, aging, hallmarks of aging, mitophagy

## Abstract

Autophagy is a major protein turnover pathway by which cellular components are delivered into the lysosomes for degradation and recycling. This intracellular process is able to maintain cellular homeostasis under stress conditions, and its dysregulation could lead to the development of physiological alterations. The autophagic activity has been found to decrease with age, likely contributing to the accumulation of damaged macromolecules and organelles during aging. Interestingly, failure of the autophagic process has been reported to worsen aging-associated diseases, such as neurodegeneration or cancer, among others. Likewise, it has been proposed in different organisms that maintenance of a proper autophagic activity contributes to extending longevity. In this review, we discuss recent papers showing the impact of autophagy on cell activity and age-associated diseases, highlighting the relevance of this process to the hallmarks of aging. Thus, understanding how autophagy plays an important role in aging opens new avenues for the discovery of biochemical and pharmacological targets and the development of novel anti-aging therapeutic approaches.

## The Autophagic Process

**Figure 1:**
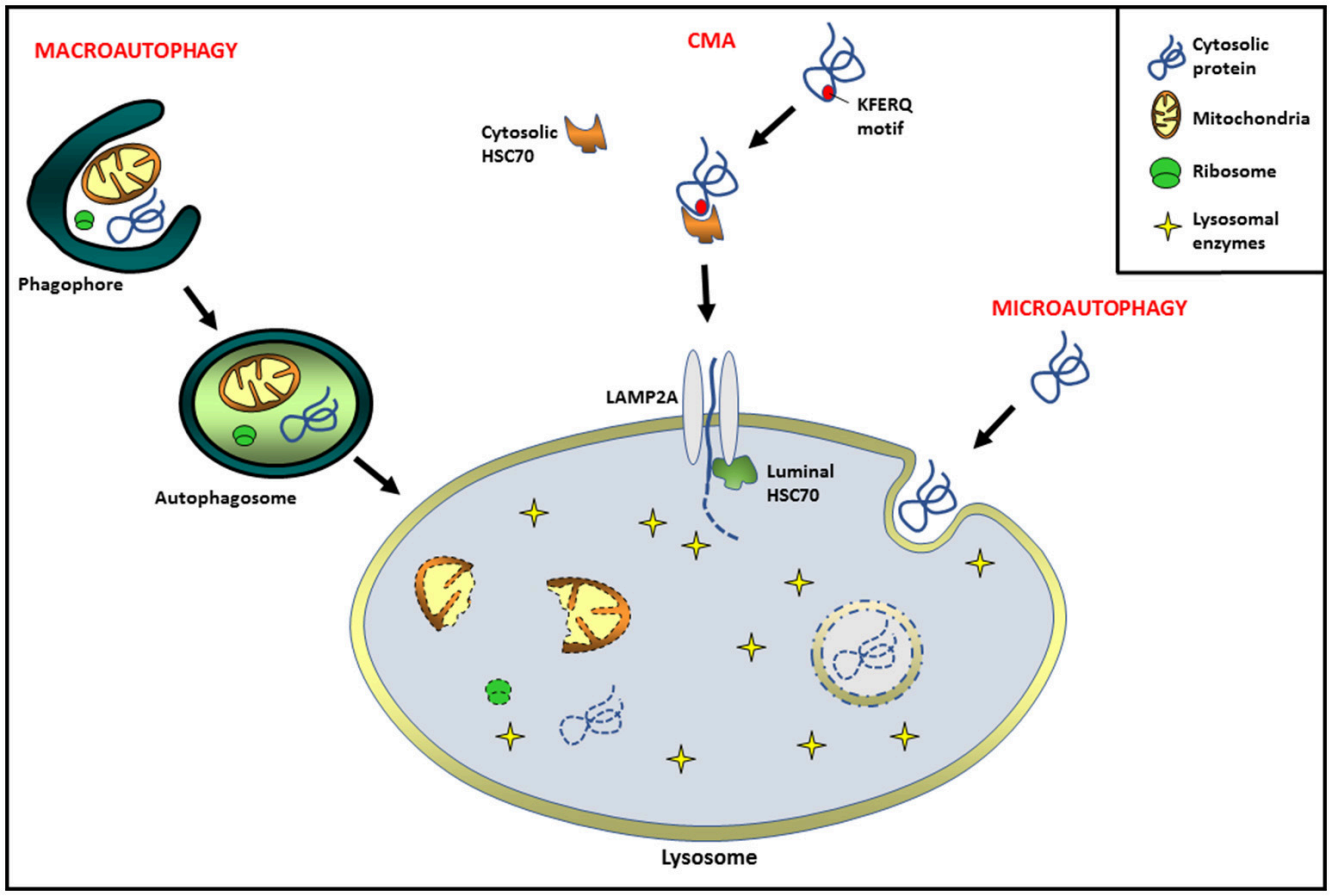
Different types of autophagy pathways in mammals. Macroautophagy: extension of a specialized membrane (phagophore) surrounds molecules and organelles, forming a double membrane vesicle called autophagosome. Finally, the fusion of autophagosomes with lysosomes leads to cargo degradation. Chaperone-mediated autophagy (CMA): proteins containing a KFERQ motif are delivered to lysosome via cytosolic HSC70 chaperone complex. The receptor lysosome-associated membrane protein type 2A (LAMP2A) is necessary for substrate translocation into the lysosomal lumen, where the degradation occurs. Microautophagy: invagination of the lysosomal membrane engulfs cytosolic cargo in small vesicles for its degradation inside.

Autophagy, literally meaning “self-eating,” is an evolutionarily conserved catabolic process in eukaryotic cells by means of which intracellular components and extracellular incorporated material are delivered into lysosomes, where their degradation occurs (1). Since its discovery, autophagy has been associated with the maintenance of cellular homeostasis, as well as the cytoplasmic quality control process (1, 2). Its dysregulation has being related to a diversity of pathological or physiological processes such as neurodegenerative, infectious, and metabolic disorders, as well as cancer and aging, among others (3–5). Several studies have demonstrated that autophagy can be very selective in targeting its cargo for degradation. Three major types of autophagy have been identified: macroautophagy, microautophagy, and chaperone-mediated autophagy (CMA). Macroautophagy (hereafter referred to as autophagy) begins with the extension of a specialized membrane, known as the phagophore, derived from the endoplasmic reticulum (ER), the mitochondria, and the Golgi cisternae (6, 7). The phagophore engulfs the molecules and organelles to be eliminated, forming a double membrane vesicle called autophagosome (7, 8). Finally, autophagosomes are targeted to lysosomes and fusion occurs, the sequestered material is degraded and released back into the cytosol (8). In microautophagy, the lysosome picks up cytosolic components directly via invagination of the lysosomal membrane (9). On the other hand, CMA is a process involving the direct transport of cytosolic components across the lysosomal membrane via chaperone proteins. Several studies have demonstrated that CMA is a highly regulated and degradative event, involving HSC70 (heat shock protein 70 complex) and multimerization of the LAMP2A receptor (lysosome-associated membrane protein type 2A). Interestingly, not all proteins are able to be CMA substrates. To undergo CMA degradation, proteins must contain a KFERQ motif in their amino acid sequences, which is necessary to bind the chaperone HSC70 (10, 11). Substrate and the HSC70 complex can bind a 12-amino-acid cytosolic tail of LAMP2A for lysosomal docking. In addition, LAMP2A multimerization is necessary for substrate translocation into the lysosomal lumen. Cytosolic HSC70 is released from the multimeric complex, and then a chaperone HSP90 (located at the lumen of the lysosomal membrane) interacts with LAMP2A, stabilizing it during the substrate translocation. Finally, a luminal chaperone HSC70 is required to end the translocation process, and once inside the targeted protein is degraded by the lysosomal enzymes (12) (Figure 1).

Autophagy can be induced by a variety of stressors, and nutrient restriction is one of the major stimuli, capable of rapidly activating the autophagic process with the concomitant inhibition of protein synthesis (1). Studies in both yeasts and mammals have characterized at least 40 autophagy-related genes (Atg), which encode proteins that participate in autophagy (13). In addition, the canonical autophagy pathway includes the inactivation of mammalian target of rapamycin complex 1 (mTORC1), allowing the phosphorylation and activation of the Unc-51-like kinase complex (Ulk1/2), with the subsequent cascade activation of the other ULK complex members such as FIP200 and ATG13 (14, 15). Another important complex that is activated is the BECLIN1, in which VPS34, one of its members, is translocated into the ER membranes and it produces high levels of phosphatidylinositol-3-phosphate, which is necessary for the recruitment of other effectors such as WIPI2b (16). Next, this effector interacts and recruits ATG16L, which binds ATG5-ATG12 conjugated to generate the ATG12-ATG5-ATG16L complex. This complex is required for the lipidation of LC3 (Microtubule-associated protein light chain 3), because it determines the site where LC3 will be conjugated and activated to LC3-II (17). Moreover, ATG3 (an E2-like protein) is associated with LC3-I and it binds to the complex through ATG12, allowing the conjugation of LC3-I with phosphatidylethanolamine to generate LC3-II. LC3-II, which is present in both inner and outer membranes of autophagosomal structures and is necessary for phagophore extension, cargo engulfment, and vesicle closure to form the autophagosome. Additionally, the targeted cargo is able to bind receptor/adaptor molecules like p62, NDP52, and NIX. These proteins contain a LC3 interacting region (LIR), which allows the recognition of elements to be engulfed by the phagophore and eliminated in an autophagic manner (18, 19).

In addition to degradation, autophagy, or part of its machinery, can mediate a regulated cell death, named autophagy-dependent cell death (ADCD). Moreover, autophagy can participate in other cell death types [reviewed in reference (20)]. Interestingly, despite the fact that regulated cell death of malignant cells is a pro-survival mechanism at the whole organism level, it can also lead to tissue degeneration and function loss, and this can reduce the fitness of the aged individual (21).

## Aging

Aging, the natural event occurring in all living organisms, can be defined as a deterioration of the cell functioning due to damage accumulation over time (22–25). This is an important biological, demographic and socio-economic issue all over the world. Dr. Barja points out that all living organisms have different longevity, indicating that evolution has played an important role in regulation and flexibilization of aging between species, in a relatively fast process (26). The understanding of the molecular basis of aging and longevity could let us manipulate it somehow in the future. In this regard, in the last 50 years numerous investigations related to aging have emerged, trying to explain this unstoppable process.

Despite the general accepted concept that aging is a multifactorial process, several theories have emerged in an attempt to explain it as a single predominant age-related change. A popular aging theory is the “Stochastic Theory,” which suggests that aging results from random damage accumulation. This can be due to external and internal sources over time, in addition to a failure of the repairing capacity. On the other hand, other theories support the idea that aging is a regulated process, mainly by the genetic code, such as the telomere length, the number of divisions that a somatic cell can go through (the “Hayflick limit”) and spatio-temporal regulation of gene expression (27, 28). Nevertheless, one of the most popular theories is the Free Radicals (or Oxidative Stress) Theory of Aging, which hypothesizes that an accumulation of Reactive Oxygen Species (hereafter ROS) falls into an oxidative damage of biomolecules, with the consequent cell functioning decline (27–29). A considerable body of evidence supports this theory, because it points to an increase in ROS cellular levels as we age, due to a higher production of them as well as a failure in the anti-oxidant systems (30, 31).

## Autophagy in Aging

Several animal models have contributed to our understanding of how the impairment of autophagy and redox homeostasis can result in age-related diseases. In the same way, numerous studies involving genetic ablation or induction of autophagic genes have revealed the importance of this process in aging of yeast, nematodes, flies, and mammals (32). The most important work that links an overexpression of a single Atg gene with an increment in mammals' lifespan was conducted by Pyo and collaborators. The authors overexpressed Atg5 in mice and found an enhancement of the autophagy process and anti-aging features, compared with the wild type mice. The mean lifespan was also incremented, suggesting the importance of autophagy in the longevity of mice (33). Another approach that demonstrates the importance of autophagy in aging has been done in Ana María Cuervo's laboratory. In aged mice, they generated a double transgenic mouse model, in which it was possible to modulate the expression of the lysosomal receptor for CMA. The results revealed that the enhancement of this receptor can prevent features of aging at cellular and organ levels (34). In addition, mice overexpressing Atg5 showed a better resistance to age-related obesity and enhanced insulin sensitivity, exhibiting an improved metabolism in aged individuals (33). Despite the mentioned studies, several others failed to demonstrate that upregulation of a single autophagic component can extend lifespan (32). Moreover, several KO mouse models have been shown to have extended lifespan, although the molecular mechanisms behind it and the connection with aging are not yet clear (35).

Notably, another relationship between autophagy augmentation and extended lifespan has been reported in exceptionally healthy centenarian humans, who have increased levels of BECLIN1, compared to young people (36). We hope that in the next years these preliminary studies in humans will be more advanced, providing insights into our species longevity mechanisms from clinical case studies.

The knockout for essential Atg genes is lethal in mice, and tissue-specific ablation has a less-dramatic phenotype, manifesting premature signs of aging (37). Specific-Atg5 or Atg7 KO leads to neurodegeneration or tissue abnormalities in most of the cases available in the literature [for a more detailed summary see reference (37)].

Finally, as we age, the incidence of cancer rises, probably because of the decline of homeostatic processes and the increase in the accumulation of potentially harmful molecules such as ROS and protein aggregates. Autophagy has been proposed to have a dual role in tumorigenesis, being important both in suppression as well as in tumor progression and surveillance (38, 39).

## ROS Generation and Aging

The ROS are considered metabolites of molecular oxygen during cellular respiration, being very reactive due to an unpaired electron (40). Mitochondria are the major ROS producers and perhaps the organelle most affected by them. In order to avoid detrimental effects of ROS, two important processes arise: Mitophagy and antioxidant system. Mitochondrial ROS can activate the autophagic pathway upon starvation by the activation of ATG4 (41), and this in turn leads to autophagic degradation of mitochondria (mitophagy) in order to reduce the ROS levels by limiting the number of mitochondria per cell (42). In addition, hypoxia and exercise can also trigger redox-dependent autophagy, suggesting that ROS might regulate the autophagic response to several stresses (43). Regarding the second process, the antioxidant system consists of several enzymes and molecules that react with ROS and neutralize them somehow, but the connection of antioxidants with lifespan is controversial (44). Notably, overexpression of a mitochondrial-targeted catalase in mice extends lifespan and reduces overall ROS, reinforcing the Free Radicals Theory of Aging in such model (45). Moreover, these mice showed a reduction in age-related pathologies (46). Additionally, Mn-superoxide dismutase (SOD2) heterozygous mice showed a life-long reduction, but surprisingly they did not have an accelerated aging phenotype. Nevertheless, this Sod2^+/−^ mice showed a higher oxidative damage to DNA and had higher cancer incidence compared with wild type individuals (47). By contrast, knockout of 17 genes involved in the antioxidant system exhibited no effect in lifespan: Only the knockout for Cu/Zn-superoxide dismutase (Sod1) resulted in a decrease in longevity and premature aging as well (48). This mouse model showed an increase in senescent markers, suggesting that the oxidative stress that Sod1^−/−^ mice suffer leads to DNA damage, promoting an irreversible state of quiescence (49). In addition, these Sod1^−/−^ mice showed an accelerated sarcopenia, manifesting muscular mass loss and altered neuromuscular junctions (50). Despite these controversial and unexpected results in mice, the relationship between antioxidants and their role in healthy or pathologic aging needs to be deeply studied in the future.

Finally, it is important to highlight that ROS have been proposed to be implicated in proliferation and survival signaling in certain conditions (42). A new concept has emerged recently in the aging field, termed “hormesis,” according to which low doses of a stressor can improve the cell response for a more detrimental condition in the future (32, 51). This could increase lifespan and cellular fitness (52). In this context, low levels of ROS can be beneficial due to the trigger of homeostatic responses, but its disproportional augmentation can lead to damage or aging (42, 53). From an autophagic perspective, an augmentation in ROS levels and a decline in mitophagy occur simultaneously, leading to aging (43, 52, 54).

## Hallmarks of Aging: An Autophagic View

In the last years, aging has begun to be seen as an active and highly regulated process (55). Age-related changes at cellular level include an increase in ROS, loss of proteostasis, genome instability, and telomere exhaustion, among others (23, 56, 57). These characteristic features of aging were termed “hallmarks of aging” by López-Otín et al. (52). In the following sections, we discuss how autophagy plays an important role in some of these hallmarks of aging, in both health and disease.

### Loss of Proteostasis

Proteostasis is one of the major functions of autophagy in normal tissues. Imbalance of proteostasis due to aging leads to protein aggregation, accumulation of misfolded proteins and in the end to cellular dysfunction, among others (23, 56, 57). Notably, carbonylation due to oxidative stress is one of the changes that leads to loss of proteostasis (44). To avoid cell death or dysfunction, numerous homeostatic mechanisms turn on, mainly autophagy (58) and the Ubiquitin-Proteasome-System (UPS). Because autophagy is considered one of the most important intracellular homeostatic processes, an alteration or deterioration of this pathway could modify the normal cell functioning, including a variety of diseases and normal cell physiology declination. Autophagosomes and lysosomes decline in an age-dependent manner in muscles (59), heart (43), and several other tissues. Moreover, CMA has also been implicated in removing oxidized and potentially dangerous proteins by direct lysosomal degradation (60).

The UPS is mostly implicated in the degradation of misfolded proteins, as well as short- and long-lived proteins by their ubiquitination. This process is achieved thanks to three major proteins that sequentially activate the ubiquitin tag (E1), transfer it to a second enzyme (E2), and finally ligate the ubiquitin tag to the target molecule (by E3 ligase), which eventually reaches the proteasome for degradation (61, 62). It is important to note that almost all regulatory proteins are substrates for this system (61, 63), and UPS declines with age [reviewed in (64)]. Interestingly, mTORC1 was found to regulate not only lysosomal protein degradation, but also proteasomal proteolysis of long-lived proteins, independently of protein synthesis, suggesting a common regulation of both proteolytic systems by nutrient-sensing (63). In addition, overexpression of a sole subunit of the proteasome enhanced its activity and the survival against several oxidants in two cell lines as well as primary culture of human fibroblasts (65). Moreover, proteasome activity decreased in an age-dependent manner (66). Overexpression of proteasome subunits in aged dermal human fibroblasts ameliorated the aged phenotype and restored the oxidized and ubiquitinated proteins to young levels (66). In the same way, transgenic mice with reduced proteasomal activity accumulated oxidized and ubiquitinated proteins, accelerating the aging phenotype and the age-related metabolic diseases (67). Besides, inhibition of proteasome activity impaired cell proliferation and shortened lifespan (68), reinforcing the importance of a correct proteostasis in healthy aging and longevity.

Several studies have been done on neurodegenerative diseases related to aging and autophagy, including those most relevant for their high impact on human population. Most of them share the accumulation of ROS, misfolded proteins, and damaged organelles, aging being the main risk factor (69, 70). This accumulation interferes with proper axonal traffic, enhancing neurotoxicity. Both autophagy and CMA impairment hamper the correct protein-aggregates degradation and the remodeling of dendrites and axons, thus diminishing the nervous plasticity (71, 72). In Parkinson's disease (PD), the cytoplasmic aggregates are formed by α-synuclein and ubiquitin (or Lewy bodies) in dopaminergic neurons of substantia nigra, leading to their death (32). Alterations in UPS (71) and also in CMA can develop the disease too. It is worth noting that overexpression of Lamp2A improved CMA performance and decreased α-synuclein cytoplasmic levels (73). In the same way, Alzheimer's disease (AD) is characterized by intracellular accumulation of tau protein as well as β-amyloids (Aβ), derived from the amyloid precursor protein (APP). This aggregate formation impairs normal cell function, finally leading to cell death (32). Also, extracellular Aβ-plaques secreted by autophagosomes can interrupt intercellular communication (72), another hallmark of aging (52). Besides, Apolipoprotein E4 (ApoE4) is the main genetic risk for sporadic AD and was found to promote the disruption of the lysosomal membrane together with Aβ, leading to neuronal degeneration (72, 74). Mutations in Presenilin1 or 2 (PS1 and PS2, two transmembrane subunits of gamma-secretase), as well as in tau protein or in APP are common causes of the familial AD (72). Other neurodegenerative diseases implicate alterations in the autophagic process, i.e., SENDA, Huntington, Amyotrophic Lateral Sclerosis, and Frontotemporal Dementia disorders [reviewed in (70, 75, 76)]. In all these cases, the lack of proper degradation by autophagy promotes the aggregation of several proteins and the consequent malfunctioning of axonal transport.

Regarding metabolic diseases and autophagy, it is well known that islet amyloid deposition leads to type 2 diabetes in humans due to the amyloidogenic property of human islet amyloid polypeptide (hIAPP). It is important to note that mice do not develop such aggregation. In order to bypass this model difference, Kim et al. developed transgenic mice expressing hIAPP specifically in β-cells and bred them with Atg7^Δβ−*cell*^ mice. Male mice had premature diabetes, while females had hyperglycemia but never developed the disease, suggesting a synergism between autophagy deficiency and human amyloid overexpression. Moreover, primary culture of monkey islet cells overexpressing precursors of hIAPP showed that autophagic inhibition by 3-methyladenine (3-MA) increased pro-hIAPP dimer or trimer accumulation, blocking the autophagic activity in these pancreatic cells (77). It is also important to highlight that diabetes or glucose handling deficiencies are risk factors for the AD, as the amyloids properties of proteins implicated in metabolic diseases and AD are similar and probably interconnected (78). More studies regarding the connection between metabolic and neurodegenerative diseases are required for a better understanding of the molecular basis of such relationships at systemic level.

Finally, sarcopenia is characterized by a progressive loss of muscle mass and strength thanks to an imbalance between production and degradation of proteins (79). Aged-related declination of autophagy (both mitophagy as well as CMA) promotes sarcopenia by protein accumulation interference with normal myofibers functioning, but an exacerbation of autophagy can also result in cellular stress and finally death (79). Thus, an age-related imbalance of proteostasis could drive a variety of diseases involving both protein accumulation and degradation.

### Mitochondrial Dysfunction

Mitophagy is a basal process involved in the autophagic degradation of mitochondria (76, 80, 81). It is necessary in normal differentiation of certain cell types such as red blood cells (82), in embryogenesis, immune response, cell programming, and cell death (80). Mitophagy is required not only to remove damaged mitochondria, but also to promote the biosynthesis of new ones, supporting the mitochondrial quality control (76, 80). Given that mitochondria are implicated in bioenergetics and ROS production, the mitophagy plays an important role in cell homeostasis. Additionally, a decrease in mitophagy is observed in aged animals and this contributes to aging phenotype (81).

**Figure 2:**
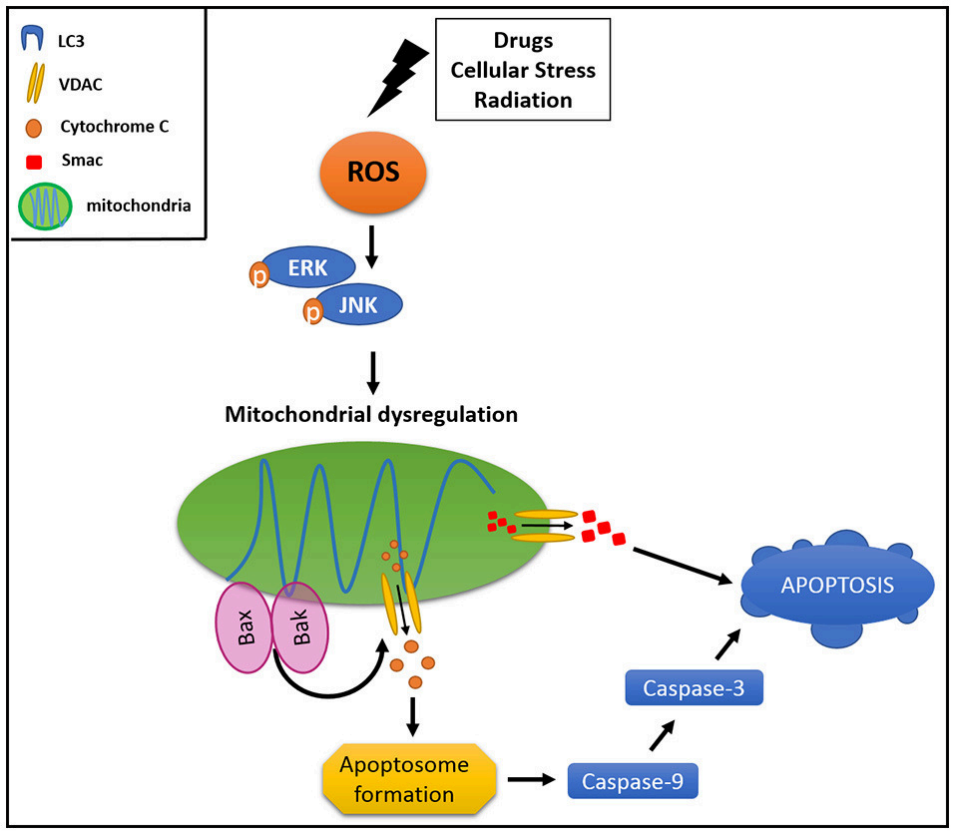
Mitophagy protects cancer cell from apoptosis. Different stimuli could drive an activation of mitochondrial dysregulation, triggering signaling pathways involved in activation of pro-apoptotic proteins (BAK and BAX). This results in MOM damage and the consequent cytochrome c and SMAC release to the cytoplasm, activating intrinsic apoptotic pathway.

Canonically, mitophagy is triggered by the cytosolic exposition of mitochondrial outer membrane (MOM) proteins, which have a LIR domain. The mitophagy is tightly regulated by several molecules, NIX and BNIP being two of the most widely characterized mitochondrial adaptors for autophagic machinery (83). NIX activation is associated with an increment in mitochondrial degradation in HeLa cells, protecting them against cellular stress (84). Interestingly, NIX has a LIR domain which binds LC3 once it is activated by phosphorylation (84). Additionally, PINK1 and PARKIN have been involved in the regulation of mitophagy when the mitochondrion loses its membrane potential (80). These proteins have been considered as key components in controlling the activation of mitophagy (85) and also as participants of mitophagy-associated cancer resistance. PINK1 and PARKIN are activated in response to an increment of intracellular ROS levels, which stimulate the MAPK and ERK1/2 signaling cascades, triggering parapoptosis in non-malignant cells, which bypass the caspases activation and, thus, the apoptosis (86) (Figure 2).

Additionally, Mitofusin 2 (MFN2) is a mitochondrial membrane fusing protein involved in several processes, including mitochondria fusion and mitophagy. Its expression declines with age, and its deficiency provokes precocious sarcopenia, accumulation of damaged mitochondria, and metabolic disorders in young mice (87). In addition, Humanin, an antiapoptotic mitochondrial protein, is capable of activating the CMA machinery, thus protecting several cell types from oxidative stress (88). Interestingly, both CMA and Humanin decline with age (89, 90), contributing to the age-related deterioration of proteostasis and mitochondrial functionality. As can be seen, several proteins regulate the mitophagy and contribute to the mitochondria homeostasis. As almost all of them decline with age, the modulation of mitophagy regulatory proteins could be a novel anti-aging therapeutic approach in the future. Despite this, more studies are needed in order to understand the complex regulation of mitophagy and the relationships between the players.

Different compounds, intracellular changes or stimuli could drive an activation of mitochondrial dysfunction. Normally, ROS oxidative stress, loss of membrane potential, MOM permeability, and aging are able to cause mitochondrial dysregulation. This imbalance triggers signaling pathways involving activation of pro-apoptotic proteins of the BCL-2 family such as BAK and BAX, resulting in an MOM damage and the consequent release of cytochrome c and SMAC (second mitochondrial-derived activator of caspase) to the cytoplasm (91), activating the intrinsic apoptotic pathway through caspase 9 (92, 93) (Figure 2). Furthermore, hypoxia inducible factor 1α (HIF1α) is able to trigger mitophagy by stabilization and activation of NIX protein, and is also responsible for autophagic activation through VMP1 (vacuole membrane protein 1) promoter, causing colon cancer resistance to photodynamic therapy (94).

Disruption in mitophagy, and thus in redox homeostasis, can produce different cardiovascular pathologies (43). PINK1-KO mice developed a decline in cardiac function due to dysfunctional mitochondria and an increase in oxidative stress (95). Notably, the same features were observed in human end-stage heart failure samples, where diminished PINK1 levels were also found (95). Despite the ubiquitous KO model, the authors were capable of demonstrating the importance of PINK1 in heart functioning. Additionally, altered mitophagy due to elevated ROS production has been linked to Alzheimer Disease (AD), but there is controversy as to whether this disturbance in the autophagic pathway is a cause or a consequence of AD (72). Remarkably, PARKIN and PINK1 have been found mutated in Parkinson Disease (PD) patients, suggesting the importance of this pathway in dysfunctional mitochondria clearance by autophagy (76, 96). In 2015, Sun et al. published a new approach for measuring mitophagy *in vivo*, using a transgenic mouse model consisting in the mitochondria-targeted overexpression of a fluorescence reporter named Keima (81). We think that this tool could be very interesting for the *in vivo* study of mitophagy and its regulation under a wide variety of conditions. Deeper studies are then required to fully understand this process and its role in healthy and pathologic aging.

### Deregulated Nutrient Sensing

Cellular response to nutrient privation implies some kind of intracellular sensor, capable of triggering the corresponding survival mechanisms. It has been proved that nutrient sensing is a highly conserved process across eukaryotes (97). Several nutrient-related signaling pathways converge on mTOR [mammalian Target Of Rapamycin; (97)], which triggers the response to growth factors, energy, glucose or amino acid changes (38, 97–100). Interestingly, nutrient sensors can also be activated under oxidative stress conditions, suggesting a common regulatory mechanism linking redox homeostasis and nutrients availability [reviewed in (101)].

The kinase mTOR is capable of linking environmental conditions with reproduction and somatic maintenance, thus influencing the individual lifespan (102). In addition, mTOR and mslt8 (a positive regulator of mTORC1) haploinsufficient female mice showed an increment in lifespan, compared to wild type mice (103). Mice carrying hypomorphic alleles of mTOR also had an increase in lifespan, a reduction in aging biomarkers, and a normal metabolism (104), showing the importance of nutrient sensing in aging. Moreover, Ribosomal S6 kinase 1 (S6K1) knockout mice showed an extended lifespan, compared with wild type counterparts, demonstrating again the importance of the mTOR pathway inhibition in longevity (105).

Besides, mTOR forms two distinct complexes, the Complex 1 (mTORC1) being capable of integrating different responses depending on nutrients availability (99, 106, 107). Upstream mTORC1, there are multiple regulating complexes, responding to each amino acid, glucose or growth factor input (99, 106, 107). Notably, p62 is one of the mTOR interactors upon amino acid stimulation, and it has been proposed to specifically stabilize the activated mTORC1 at the lysosome surface (108). It is well known that p62 is an autophagic adaptor protein whose role in nutrient sensing pathway might be another connection between mTOR and the regulation of autophagy.

Because autophagy is a catabolic mechanism, it can be assumed to be implicated in cellular and systemic metabolism. Metabolic stress responses could be compromised due to a decline in autophagic activity (109). As an important process regulating the general cellular status, autophagy can also crosslink metabolic pathways to maintain the homeostasis under a variety of conditions (43). In this sense, it has been demonstrated that, after nutrient or growth factor deprivation, ULK1 and ULK2 are activated, and these kinases phosphorylate and activate several glycolytic enzymes as well as autophagic proteins. This makes it possible to obtain metabolites thanks to glucose uptake, gluconeogenic pathway blockage, and autophagic degradation of cytosolic components (110). Supporting this, mTOR hyperactivation was found in several diseases such as obesity, metabolic syndrome, and type 2 diabetes (100), which highlights the importance of a tight regulation of autophagy as well as the nutrient sensing pathway.

Given that mTOR is capable of sensing the nutritional state of the cell, it was proposed to play an important role in Caloric Restriction (CR) therapy. Indeed, mTOR signaling network was shown to mediate lifespan extension by CR. Notably, the sole amino acid-restriction is enough to promote CR-response (111). Sirtuins 1 and 3 (NAD+ deacetylases) are activated in response to CR, as well as SOD1, in order to change the metabolism accordingly and prevent oxidative damage, respectively. Such ROS regulation rules out the Free Radicals Theory of Aging, at least partially (101, 112, 113). It is important to highlight the fact that inhibition of mTOR (specifically mTORC1) in embryogenesis is lethal, while the ablation of these pathways in adulthood can extend life [reviewed in (111)]. In fact, weight loss is suggested as prevention as well as therapy for a variety of age-related diseases (98).

### Genomic Instability

In the last decade, several studies have demonstrated that autophagy or autophagic-related molecules act as a “safeguard” of genome stability both directly (DNA repair modulation) and indirectly (by acting as a homeostatic response) (114). Several mouse models have provided substantial information regarding genomic instability and its connection with healthy and pathological aging (55).

Regarding oxidative stress and DNA damage, ROS increase is thought to be mainly harmful for the mitochondrial DNA (mtDNA), generating the mutagenic 8-hydroxy-20-deoxyguanosine (8-OHdG), as well as mutations and deletions in mtDNA that result in a dysfunctional mitochondrion (69). Moreover, mitochondrial dysfunction promotes telomere attrition, telomere loss, and chromosome alterations, culminating in apoptosis in mouse embryos (115). Besides, Donati and collaborators demonstrated that, upon autophagic stimulation with an anti-lipolytic agent in 16-month-old rats, 8-OHdG accumulation in liver was successfully supressed, reaching the values obtained from young animals in only 6 h. When they measured the cytochrome c oxidase activity, they found that this decrease was not associated with lower mitochondrial enzyme activity, demonstrating the selective mitophagy of a small population of 8-OHdG-positive mitochondria and the importance of this proteostatic process in anti-aging mechanisms (54, 116). In the same way, dietary restriction reduced 8-OHdG levels in mitochondrial DNA (mtDNA) of aged rats and mice compared with those fed *ad libitum* (117), supporting the importance of dietary restriction in prevention of mtDNA damage by ROS in aged animals. Notably, Sod2^−/−^ mice accumulated high levels of 8-OHdG both in nuclear and mitochondrial DNA, compared with wild type mice. Nevertheless, they showed no changes in lifespan or age biomarkers (47). On the other hand, Atg7^−/−^ mouse keratinocytes presented premature aging after oxidative stress induction, supporting the importance of autophagy in healthy aging (118). In addition, Bender et al. found high levels of mtDNA deletions in dopaminergic neurons of PD patients, compared to controls (119). As we have already mentioned, PARKIN and PINK1 are mutated in PD, thus altered mitophagy can explain, in part, the accumulation of mtDNA damage in PD patients.

Autophagy has emerged as an important process in genome maintenance. After treatment with several cell cycle blockers, human osteosarcoma cells (U2OS) increased the micronuclei frequency as well as autophagosomes. Importantly, the authors observed a small but significant colocalization between them. Knockdown of Atg5 or Atg7 abolished this colocalization. P62/SQSTM1 also colocalized with micronuclei, indicating that micronuclei can be degraded by autophagy and this may contribute to genome stability (120). Moreover, NDP52 and p62-dependent autophagy can degrade retrotransposon RNA, preventing new insertions into the genome of long and short interspersed elements (121). Additionally, autophagy deficiency leads to an accumulation of RHOA with p62. This phenotype drives cytokinesis failure, aneuploidy, and multinucleation due to inappropriate formation of contractile ring (122). Furthermore, allelic loss of Beclin1 promotes tumorigenesis and activation of DNA-damage response in neoplasic cells. In this context, autophagy deficiency leads to genome instability under metabolic stress in these mouse mammary epithelial cells (123). Artificially aneuploid mouse cells showed increased autophagy to protect cells from genome instability (124).

Autophagic adaptor p62 has been found to be implicated in genome instability in several studies. Accumulation of p62 led to the activation of DNA-damage response (125). By contrast, overexpression of p62 (or autophagy deficiency) suppressed DNA-damage response by its direct inhibitory interaction with RNF168, an important E3 ligase for histone H2A ubiquitination and DNA-damage response (126). In this regard, p62 downregulates the protein levels of several molecules involved in homologous recombination (HR) of damaged DNA, inducing at the same time non-homologous end-joining (NHEJ), stressing the importance of p62 nuclear accumulation upon several stresses (127). More studies are needed to completely elucidate the role of p62 and other autophagic components in genome maintenance throughout life.

### Epigenetic Alterations

Epigenetic changes due to external or internal factors drive several processes, including development and aging (128). In muscular and hematopoietic stem cells, different histone modifications help to establish the quiescence state with age (129). Besides, epigenetic alterations lead to different responses in aging and longevity in several mouse models [reviewed in (55)]. Notably, macrophages of old mice had hypermethylated LC3 and Atg5 promoter regions, compared with those of young mice, downregulating the protein levels and promoting aging decline of autophagy (130).

The natural polyphenol resveratrol, an autophagic inducer, has been proved to exert its action by inositol 1,4,5-triphosphate receptor (IP_3_R) and by protein acetylation decrease (131, 132). In this sense, a balance between different degrees of protein acetylation is presumed to be important for autophagy (32). High levels of acetyl-CoA, which serves as an acetyl group donor, were found to promote histone hyperacetylation, downregulating the expression of Atg genes, thus inhibiting autophagy and shortening lifespan in aged yeast (133). In addition, these authors found that the knockout of a certain acetyl-CoA in *Drosophila melanogaster* brain enhances autophagy and prolongs lifespan. Morselli et al. used specific siRNA knockdown of Sirtuin1, a NAD^+^ deacetylase in HCT116 cell line, which resulted in a suppression of the autophagic response to resveratrol. Importantly, resveratrol induced autophagy by AMP-dependent kinase/mTOR-independent pathway and changed the acetylation of 375 proteins, half of them involved in autophagy. On the other hand, deacetylations were often observed in metabolism-related proteins, thus activating autophagy (131). Another autophagic inducer, spermidine, is a well-known pro-longevity polyamine (56), largely studied as an anti-aging agent. Like resveratrol, spermidine can induce both acetylation and deacetylation changes that promote the autophagic pathways via AMP-dependent kinase signaling. In this regard, low doses of this polyamine together with low doses of resveratrol synergistically caused the same autophagic response as higher doses of each inducer separately (131).

Taken together, organismal models as well as *in vitro* studies highlight the importance of epigenetics throughout life. The relationship between epigenetic changes and autophagy needs to be deeply studied in order to understand the regulatory loop that seems to be involved in development and aging.

### Telomere Attrition

Telomeres are specific repetitive sequences at the end of chromosomes, the telomerase is the special polymerase involved in the elongation of this protective zone. It is important to note that somatic cells of mammalian species lack the expression of telomerase. Thus, telomerase is highly regulated in a spatio-temporal manner (134). In each cell cycle, the telomere becomes shorter and shorter, leading to a vulnerability of the chromosomal end. This process is called telomere attrition and is considered another hallmark of aging (52).

Telomere shortening can cause genomic instability, and thus contributes to cancer or age-related diseases (115). This progressive diminishment in telomere length can influence gene expression and provoke several pathologies without damaging DNA (134). Moreover, ROS can trigger telomere attrition or loss and genomic instability, which can be prevented by using an antioxidant (115). In addition, telomerase activity can support cell cycle progression by preventing the arrest due to short telomeres, leading to a putative malignancy. Remarkably, overexpression of Beclin1 in HeLa cells revealed that telomerase activity is reduced after autophagy induction (39). This approach argues in favor of the hypothesis that autophagy plays an important tumor suppressor role by the modulation of telomerase activity in somatic cells. Similarly, Guanosine-rich zones, like telomeres, can suffer the formation of G-quadruplexes, interfering with gene expression and cell growth (135). In this regard, melanoma cells treated with an anthracene-based ligand (Ant 1,5, capable of stabilizing G-quadruplexes) showed an increase in genome instability and telomere dysfunction. Furthermore, Ant 1,5 induces a p21-dependent augmentation of autophagy levels. This autophagic response arises in order to avoid genome instability and telomeric dysfunction, thus promoting cell survival (135).

### Other Hallmarks of Aging

Cellular senescence is considered another hallmark of aging (52). Senescence can be induced by DNA damage, telomere attrition or other stress signals, with the consequent cell cycle arrest. Several mouse models have contributed to the current knowledge of senescence and its characteristics (55). For example, Apolipoprotein E (ApoE) knockout mice present atherosclerotic plaques, which can be prevented by the addition of rapamycin. Furthermore, autophagy regulates the senescence of vascular smooth muscle cells (VSMCs) of ApoE^−/−^ mice, involving the mTORC1/ULK1/ATG13 pathway in atherosclerosis progression (101). In addition, senescent MEF cells accumulate copper due to higher import and lower export, enhancing antioxidant defense mechanisms. In addition, rapamycin treatment can prevent and reverse copper accumulation, suggesting that autophagy mediates the copper homeostasis (136). Along the same lines, primary cultures from human fibroblasts depleted for Atg7, Atg12, or Lamp2 showed cell cycle arrest and high levels of SA-β-Gal staining, a characteristic feature of replicative senescence cells (137). Intriguingly, autophagy can mediate the transition to a senescent phenotype in IMR90 human diploid oncogene-induced senescence fibroblasts, making possible the protein remodeling needed to establish the senescent phenotype under oncogene activation (138). An interesting review conducted by Kwon et al. put forward a “toolkit” of differential diagnosis to resolve the apparent contradiction of autophagy in the cellular senescence. They proposed that type of autophagy, the exact moment when it acts, and the place where it occurs can define the pro or anti-senescence role of autophagy (139).

Another hallmark of aging, according to López-Otín et al., is stem cell (SC) exhaustion, with the consequent decline in tissue regenerative potential (52). Self-renewal is important to maintain the population of tissue-specific stem cells throughout life. Importantly, as we age, stem-cell activity decreases (140). In addition, SCs function is highly regulated in response to external stimuli (129). Ho et al. have shown that autophagy is necessary for preservation and quiescence of hematopoietic stem cells (HSCs). The authors demonstrated, in an Atg12-KO mice model, that autophagy-defective HSCs resemble old HSCs, in terms of accumulation of mitochondria, augmentation of myeloid-to-lymphoid ratio, diminishment of the regenerative potential, and decrease in self-renewal (141). This study supports the importance of autophagy in blood system homeostasis (142). Autophagy is also important to maintain stemness in bone marrow-derived mesenchymal stem cells (BMMSCs), and induction with rapamycin restores the biological properties of BMMSCs (143). In addition, Atg7 loss in aged muscle stem cells (satellite cells) of transgenic mice caused altered mitophagy and an accumulation of ROS, all features of senescence that diminish the regenerative potential of aged satellite cells (144). It is important to note that autophagic modulation could be an interesting therapeutic approach to prevent stem cell senescence and decline. Due to SC complexity, more studies are required to fully elucidate the role of autophagy in maintenance of stem cells.

## Current and Future Autophagic Treatments in Aging and Age-Related Diseases

It can be presumed that induction or restoration of autophagy and antioxidant cellular systems could alleviate aging symptoms. Three major anti-aging therapies were evaluated over the last 30 years: Autophagic inducers, antioxidant (polyamine-rich) consumption, and caloric restriction.

Rapamycin, an immunosuppressive macrolide, is a well-known autophagic activator via mTORC1 inhibition of the mTOR complex. This pharmacological treatment has proved to increase lifespan in flies, nematodes, yeast, and mice [reviewed in (113)]. Moderate doses of this drug can alleviate atherosclerosis, achieving the same effects as higher concentrations (101). Furthermore, rapamycin added during reperfusion after heart infarction in a C57 myocardial ischemia mouse model improved the survival and cardiac functioning, reducing the infarcted zone size as well as apoptosis post-ischemia/reperfusion. In addition, AKT phosphorylation increased after treatment, suggesting that AKT-ERK pathways were selectively activated by rapamycin (145). Finally, rapamycin improves whole metabolism in several ways, strengthening the importance of regulating autophagy activity by external compounds in order to ameliorate metabolic diseases at cellular and whole organism levels (100, 109).

In addition, exogenously administered spermidine extends lifespan in mice treated throughout life or at pre-aged adulthood, and in hypertensive rats as well. In addition, spermidine dietary intake was inversely correlated with cardiovascular pathologies in a human population-based cohort (146). Spermidine, thus, has been proposed to be a neuro and cardioprotector in aging models and humans, highlighting the importance of polyamine-rich diets (56).

Caloric restriction (CR) is the reduction of total calorie intake by 30-40% without malnutrition. There is strong evidence supporting this therapy as one of the most effective in reducing oxidative stress in rats and mice, prolonging lifespan (31, 113). CR has been found to provoke a decrease in ROS generation (69) and a diminishment of DNA damage (31). Regarding CR and autophagy, it was demonstrated that 8%-CR in combination with exercise or 8%-CR alone were capable of upregulating Atg7, LC3, and LAMP2 in type II skeletal muscle in rats (147). The authors also found that aging augmented BECLIN1 protein expression and oxidative stress, but CR alone or with exercise diminished this modification as well as the apoptotic index, both correlating negatively with LAMP2 gene expression (147). Sod^−/−^ mice treated with CR attenuated the age-related-like phenotype of this knockout in terms of DNA damage, cellular senescence, and inflammation (49). CR could be beneficial to human health, according to epidemiological studies (37). As CR regulates several pathways, more integrative studies are required in order to fully understand its anti-aging effect.

Finally, several reviews and research studies highlight the importance of autophagic modulation as an anti-aging therapy for the future (32, 37, 43). Rapamycin, resveratrol, polyamines, and CR are possible candidates to be tested more carefully in order to improve the putative treatments for human age-related diseases.

## Concluding Remarks

**Figure 3:**
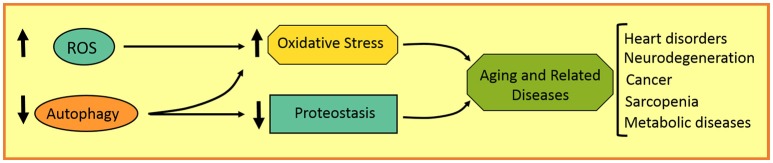
Schematic representation of aging-related disorders in autophagy and redox imbalance.

Aging involves several features that can promote the development of a variety of disorders in aged individuals, such as neurodegenerative, heart, and metabolic diseases, as well as cancer. These age-related characteristics involve a gradual increase in ROS production and genome instability, and a progressive decrease in antioxidant, DNA repairing, and proteostatic systems, among others (Figure 3). Autophagy, as a homeostatic process, plays an important role in the maintenance of cell physiology and avoidance of any internal or external damage that could eventually appear. Several attempts were made to improve age-related features, such as caloric restriction as well as antioxidants and autophagy inducers. Rapamycin, resveratrol, and polyamines are autophagic inductors clinically available that could improve aging and some age-related disorders. It is important to highlight the fact that chemotherapies combined with autophagic inhibitors (i.e., chloroquine and derivatives) could be more effective in cancer treatment. Further studies are required to make autophagy modulation a more promising anti-aging and anti-tumoral therapy in the next decades.

## Author Contributions

MB analyzed the data, wrote the manuscript, and critically read the manuscript. RG wrote the manuscript. CF designed the research, analyzed the data, wrote the manuscript, produced the figures and critically read the manuscript.

### Conflict of Interest Statement

The authors declare that the research was conducted in the absence of any commercial or financial relationships that could be construed as a potential conflict of interest.
